# Evaluation of an innovative plastic cube phantom designed to improve the efficiency of accelerator QA

**DOI:** 10.1120/jacmp.v1i4.2637

**Published:** 2000-09-01

**Authors:** J. C. Huang, L. E. Reinstein

**Affiliations:** ^1^ Department of Radiation Oncology Winthrop University Hospital New York 11501; ^2^ Department of Radiation Oncology State University of New York at Stony Brook Stony Brook New York 11794

**Keywords:** phantom, reproducibility, charge effect

## Abstract

A new cubic phantom was designed to improve the efficiency on the QA measurement of accelerator. It has a variety of applications, such as dose constancy check, depth dose verifications, and symmetry and flatness evaluations. In particular, this new design makes it much easier to perform the check on output constancy vs. gantry angles as a cylindrical ion chamber positioned at the center of the phantom. The reproducibility of the setup using this phantom has been investigated. The charge effect of the phantom is found to be insignificant. It also reduces the monthly dosimetric QA time spent on a typical multimodality accelerator by approximately 40%.

PACS number(s): 87.66.–a

## I. INTRODUCTION

AAPM TG40 protocol^1^ recommends monthly output and depth dose constancy tests of photon and electron beams, which typically require ionization chamber measurements. These measurements are routinely done in a solid phantom instead of a water phantom for practical reasons. Since a modern accelerator may have dual photon beams, plus five to eight electron beams, an ion chamber would be placed at 20 or more different depths during a typical monthly QA procedure. These measurements are time consuming since it can be awkward to position an ion chamber precisely inside a set of traditional plastic slabs.

In order to simplify and to expedite the energy and output constancy QA measurements, a new cubic phantom was designed.^2^ ^,^ ^3^ It allows an ion chamber to be placed at different depths with a high degree of accuracy and reproducibility without re‐adjusting the phantom's lateral position or vertical height. Using the new phantom, the time spent for monthly QA measurements is reduced to approximately two‐thirds of the usual time period. Furthermore, the output and the flatness and symmetry of the beams can be easily checked at three orthogonal gantry angles. Since density and other physical characteristics of the plastic are different from the water, the dose output or percentage depth dose measured in plastic material may not be the same as that in water. The advantages using the cubic phantom are to improve the efficiency of QA constancy measurement, but not for calibration of the machine.

## II. METHODS AND RESULTS

**Figure 1:**
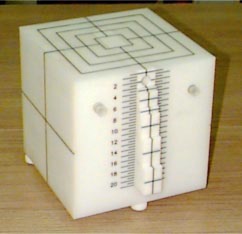
(Color) A photo of the newly designed plastic phantom.

The photo of the cube phantom is shown in Fig. 1. It is constructed from white acrylic with a nominal specific gravity of $1.194\;\text{gm}/{\text{cm}}^{3}$. The phantom has an overall physical size of $25\times 25\times 25\;{\text{cm}}^{3}$. It has eight small “domino” shaped inserts which can slide smoothly in or out, with thicknesses of 0.5 cm, 1 cm, 2 cm, 2.5 cm, 4 cm, and 5 cm, respectively. The insert with thickness of 2.5 cm is specially manufactured with a hole to hold a cylindrical Farmer ionization chamber.

The hole of a diameter of 1.5 cm is off‐centered, with a 1 cm distance to one side and a 1.5 cm distance to the other. By interchanging the order of these inserts, the ion chamber can be quickly and precisely positioned at any depth from 1 cm to 20 cm in 0.5 cm increments. The exact chamber depth can be appreciated using a block‐ruled scale (Fig. 1).

### A. Setup and measurement repeatability comparison

As a comparison, the complete monthly QA dosimetry measurements are performed using either the plastic cube phantom or a set of polystyrene slabs (SCRAD phantom) for a Varian CL20 LINAC which has one photon beam and five electron beams. All measurements are done during the same session to minimize the variation on the machine's output. A Keithley 616 electrometer is used to record the measurements. The readings of the electrometer (unit: $10^{-8}\;C$) are repeated three times, then averaged after temperature and pressure corrections. The time spent to complete the measurements is also recorded. In order to evaluate the repeatability, each set of measurements is again performed three times, i.e., the chamber is disconnected, the phantom is removed, and then the entire setup procedure is repeated. Table I shows the comparison of the output constancy measurements using both plastic cube phantom and a set of polystyrene slabs. As shown in Table I, the percentage error of the measurement using the plastic phantom varied from 0.02% to 0.15% which is better than the result using a set of polystyrene slabs.

**Table I:** The comparison of reproducibility of output constancy using the plastic phantom and a set of polystyrene slabs for 10‐MV photon beam and 6, 9, 12, 16, and 20 MeV electron beams (Varian CL20). SSD=100 cm, filed size=10×10 cm (or 10×10 cone).

Beam	15 MV	6 MeV	9 MeV	12 MeV	16 MeV	20 MeV
Depth	3.0 cm	1.0 cm	1.5 cm	2.0 cm	2.0 cm	2.0 cm
% Error using cubic Phantom	0.07%	0.06%	0.04%	0.15%	0.02%	0.03%
% Error using slabs	0.28%	0.15%	0.48%	0.21%	0.46%	0.48%

### B. Repeatability of energy constancy measurements with different combinations of inserts

**Figure 2:**
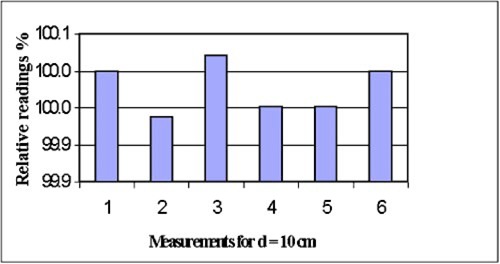
(Color) Repeatability with six different combinations of the inserts for 10‐MV photon beam (Varian CL20), all of which yield a chamber depth of 10 cm. The measurements are normalized to the first reading.

As a check on photon and electron beam energy constancy, the measurements are taken at depths greater than $d_{\max}$. Each new depth can be achieved with a different combination of plastic inserts. For example, a depth of 10 cm can be obtained by positioning the inserts from the surface to the measurement point as (1+2+2+4+1) cm. However, other possible combinations can also yield the depth of 10 cm, e.g., (4+2+2+1+1) cm, (2+2+4+1+1) cm, (2+5+1+1+1) cm, etc. To further demonstrate the repeatability of the phantom, the depth dose measurements are repeated using several different combinations, and the results are shown in Fig. 2, where the relative depth doses were normalized to the first combination of the inserts, and the index refers to the measurements with different combinations of the inserts. As shown in Fig. 1, the variation on depth dose measurements with a different combination of inserts is only 0.08%, which indicates that the order of the insert's combination is not a major concern.

### C. Charge effect

**Figure 3:**
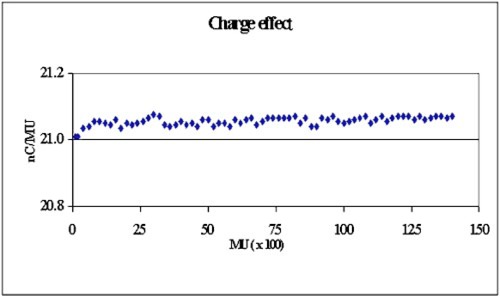
(Color) Measurements of charge effect in a 6‐MeV electron beam (Varian CL20).

When a plastic phantom is used for electron beam dose measurements, the material of the phantom is thick enough to stop electron beam and the electron charges will be stored inside the phantom then slowly leak away. This phenomenon is known as charge effect.^4^ ^–^ ^7^ Due to the charge effect, the electrometer reading will be higher if the phantom has been pre‐irradiated to a high dose with an electron beam. The charge effect of this plastic cube phantom is studied using a 6‐MeV electron beam. The chamber is placed at the depth of 1 cm and a series of 200 MU irradiation is delivered to a total of 14 000 MU. Figure 3 shows the variation of the readings per monitor unit (nC/MU) to be less than 0.5%.

### D. Off‐center ratio and output vs. gantry angles

AAPM TG40 protocol recommends checking the off center ratio (OCR) vs. gantry angle and output vs. gantry angle annually. This task is not easy to perform if using a set of polystyrene slabs (SCRAD phantom) and the setup error which is often large. To make the above task easier, in this cubic phantom, two additional holes are manufactured at the depth of 5 cm and off‐axis 8 cm, as shown in Fig. 1. It allows user to measure OCR in three orthogonal gantry angles as the phantom is turned over ±90°. The results of the OCR measurements in three orthogonal gantry angles are listed in Table II. The variation of OCR vs. gantry angles is less than 0.5%.

**Table II:** The off‐center ratio measurement for 10‐MV photon beam of Varian CL20, where beam down is at a gantry angle of 180°. Measurements are taken at SSD=100 cm, filed size=20×20 cm, depth=5 cm.

OCR\Gantry angles	180°	90°	270°
RT, off center 8 cm	1.016	1.018	1.016
LT, off center 8 cm	1.013	1.012	1.014

**Figure 4:**
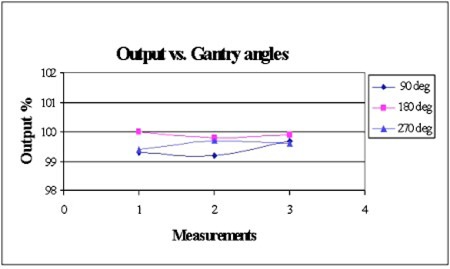
(Color) Output percent vs. gantry angles for 10‐MV photon beam (Varian CL20). There are three sets of output at gantry angles of 180°, 90°, and 270°. Data are normalized to the first set of the reading at 180°.

The comparison of output at different gantry angles of 180°, 90°, and 270° can be easily done in the cubic phantom while an ion chamber was positioned at the center of the cube with a depth of 12.5 cm to each surface. The measurements can be done without repositioning the ion chamber and flipping solid slabs as one normally does. Figure 4 shows the results of the output for three gantry angles. The variation of these measurements is found to be less than 0.8%.

To further investigate the possible effect to the dose output when beam incidents from different side of the phantom, the measurement was repeated with cubic phantom turned ±90° while gantry was kept at 180°. The variation of the output measurement at three different cube orientations was found to be less than 0.2%. Therefore, the anisotropic effect caused by beam incidence from the different side of the phantom is negligible.

## III. DISCUSSION AND CONCLUSIONS

The plastic cube phantom provides rapid, accurate, and reproducible ion chamber positioning. It has a clear and unambiguous depth readout scale in order to expedite several of the TG‐40 recommended monthly QA procedures. The results reported in this paper show that the repeatability of the QA data generated using the plastic cube for output and energy constancy is found to be better than that with the conventional plastic slab type (SCRAD) phantom. It also provides a means for beam uniformity testing using a single ion chamber. In addition, it allows precise comparison of beam output at orthogonal gantry angles, which is a difficult test using traditional stocked slab phantom. It is particularly important to note the savings of time for the QA procedures. It takes only 40 min with the plastic cube to complete a set of measurements of output constancy and depth dose constancy (for the modalities and energies described in Table I), while the same measurements take 65 min with the conventional phantom. This presents substantial potential time savings over the course of a year, especially for multiaccelerator clinics.

## ACKNOWLEDGMENTS

We wish to thank Nuclear Associates for their cooperation in this study.
